# Removing singular refractive indices with sculpted surfaces

**DOI:** 10.1038/srep04876

**Published:** 2014-05-02

**Authors:** S. A. R. Horsley, I. R. Hooper, R. C. Mitchell–Thomas, O. Quevedo–Teruel

**Affiliations:** 1 Department of Physics and Astronomy, University of Exeter, Stocker Road, Exeter, EX4 4QL, United Kingdom; 2 School of Electronic Engineering and Computer Science, Queen Mary University of London, London E1 4NS, United Kingdom; 3 School of Electrical Engineering, KTH Royal Institute of Technology, SE-10044, Stockholm, Sweden

## Abstract

The advent of Transformation Optics established the link between geometry and material properties, and has resulted in a degree of control over electromagnetic fields that was previously impossible. For waves confined to a surface it is known that there is a simpler, but related, geometrical equivalence between the surface shape and the refractive index, and here we demonstrate that conventional devices possessing a singularity — that is, the requirement of an infinite refractive index — can be realised for waves confined to an appropriately sculpted surface. In particular, we redesign three singular omnidirectional devices: the Eaton lens, the generalized Maxwell Fish–Eye, and the invisible sphere. Our designs perfectly reproduce the behaviour of these singular devices, and can be achieved with simple isotropic media of low refractive index contrast.

There is a long history of using geometry as a design tool for controlling the propagation of waves, which goes back at least as far as Fermat's principle. The development of transformation optics (TO) has fully established the relationship between geometry and material properties, and has prompted a revived interest in this area^1^,^2^,^3^,^4^. TO has been applied to design devices for electromagnetic concealment^1^, illusion optics^5^, and light harvesting^6^, all working—in principle—perfectly for waves. If we relax the requirement that a device operate perfectly for waves, and instead simply require it to be accurate in the limit of geometrical optics, further possibilities arise. Well known designs include; Luneburg's lens^7^, which focuses an incident beam of parallel rays to a point; Eaton's lens^8^, which retroreflects a beam of parallel rays incident from any direction; and the invisible sphere^9^, that sends the incident rays in a full loop so that they leave the device as if the occupied region was empty space. Unfortunately, several of the aforementioned devices exhibit an infinity at the centre of their refractive index profiles, and only imperfect truncated versions can be fabricated. A TO based technique has been developed to overcome this problem, named transmutation^10^. The resulting designs require highly anisotropic material properties, but exhibit exactly the same functionality as the original^11^,^12^,^13^. While this is a promising advance, it is often difficult to implement the required anisotropy.

While in three dimensions singular profiles can only be mimicked using highly anisotropic media, for a wave propagating on a surface we have an additional degree of freedom if we allow the surface to be deformed. Although the idea of deforming the surface into a particular shape to construct ‘geodesic lenses’ has been well established for some time^14^, in this article we demonstrate a new method where the shape of the surface is used in combination with the local refractive index to make otherwise unphysical optical devices practical. We interpret this as a technique that is analogous to the aforementioned transmutation procedure, but with the added advantage that the required materials are isotropic and low contrast. It is found that, through modifying the shape of the surface, one may manipulate the rays in a way that would require points of infinite refractive index on a flat surface. This means that it is possible to reproduce the propagation characteristics of devices with singular material properties through utilising surface curvature and finite refractive index profiles, allowing one to implement, in two dimensions, devices that were previously practically impossible to realise in three. The theory we develop is valid for any such waves, be they electromagnetic waves on surfaces^15^,^16^,^17^ or in geodesic waveguides^18^; or acoustic waves travelling on surfaces^19^ or through sheets^20^. We demonstrate the principle with three singular devices: the Eaton lens^8^, the Invisible sphere^9^ and the generalised Maxwell fish eye^21^.

## Methods

Consider waves confined to a plane that has a local refractive index of the form, 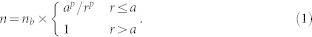
where *p* < 1, and *a* is the radius of the device (polar coordinates *r*, *θ*). The value of the background index, *n_b_* = 1 is a matter of choice, as the ray trajectories are unaffected if we multiply the index everywhere by a constant. Although the optical path to the centre of such an index profile is finite, the arbitrarily large values for the index present an obstacle in any practical implementation. Yet for waves propagating on a surface we have another degree of freedom, which is the shape of the surface. We now show that the index profile given by (1) has the same effect on rays as a deformation of the flat surface into the shape of a cone.

In order that the shaped surface mimic the planar device (1), we use the method of Tyč and Šarbort^21^, previously applied to construct surfaces for visualizing the function of spherical lenses. The shape of the surface is defined in terms of its height, *z*(*R*), and the position on the surface is specified using polar coordinates *R*, *θ*. For the surface to mimic the planar device, the optical length elements must be everywhere equal, 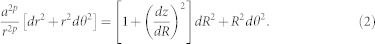
Beyond *R* = *a* the height of the surface, *z*, equals zero. Equating the coefficients of *dθ*, the above equation leads us to the following relationship between the two radial coordinates, 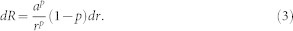
Applying (3) to (2), we obtain the condition on the slope of the surface required to mimic the refractive index profile (1), 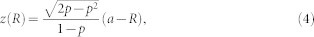
which describes a cone of angle, *α* where cot(*α*/2) = (2*p* − *p*^2^)^1/2^/(1 − *p*). Therefore, so long as the index (1) diverges slower than 1/*r* at the origin, it's effect on incoming rays confined to the surface can be mimicked through deforming the flat plane into a cone. The angle of the cone is related to how quickly the index diverges approaching the origin, and the singular point in the refractive index corresponds to the point of infinite curvature at the tip of the cone.

We can use this relationship to remove singularities from some existing designs of planar devices. For example, the Eaton lens is a device that acts as an omni–directional retro–reflector for waves^8^,^11^, and is singular at the point *r* = 0, 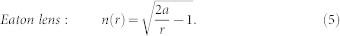
The index (5) can be split into a singular prefactor, 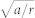 multiplied by 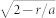. The singular part can be mimicked using a cone of angle 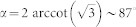. Given that *R* and *r* are related by 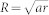 (to see this, integrate equation (3)), the effective index of an Eaton lens (5) can be implemented for surface waves using such a cone, with a layer of index 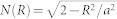 placed on its surface. We then reach the interesting conclusion that the function of a planar Eaton lens for surface waves can be obtained through deforming the flat surface into a cone upon which we place the index profile of a Luneburg lens.

A similar procedure can also be applied to other devices. We consider two further examples; the generalized Maxwell fish eye^9^,^22^, and the invisible sphere^9^, 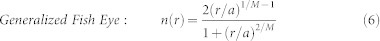

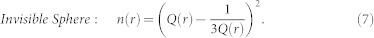
In (6) *M* is some number greater than unity that determines the function of the device, and in (7) the quantity *Q*(*r*) is given by, 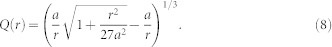
Following the same procedure used for the Eaton lens, we find the angle of the cone necessary to perform the function of the generalized Maxwell fish eye is 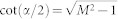, upon which we place the refractive index profile, *N*(*R*) = 2/(1 + *R*^2^/*a*^2^). The generalized fish eye is thus equivalent to the Maxwell fish eye profile on the surface of a cone. A modification of the slope on this cone will produce all of the possible lenses offered by the Maxwell Fish Eye profile. Finally, we find that the invisible sphere is equivalent to a cone of angle 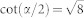 upon which we place the index *N* = (*R*/*a*)^2^[*S* − 1/(3*S*)]^2^, where *S* is given by (8), with *r* = *R*^3^/*a*^2^. Although the cone required to mimic the invisible sphere is quite sharp, the index profile ranges only up to a value of ~1.59.

**Figure 1:**
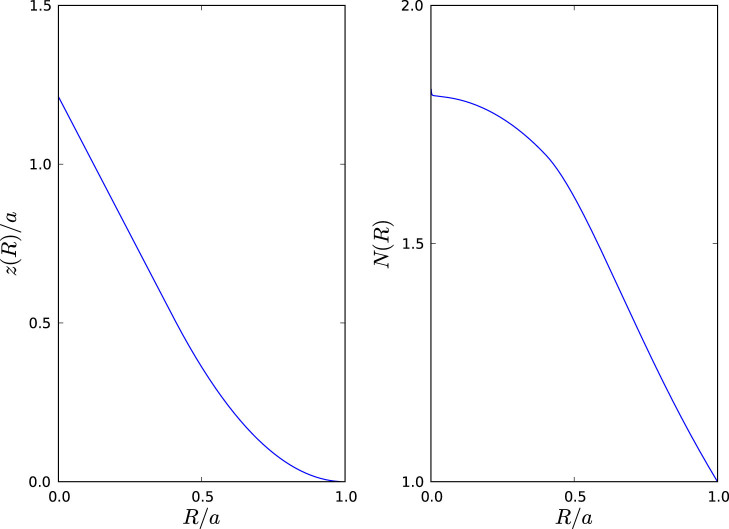
Surface shape and index distribution. The left plot shows a smoothed surface profile *z*(*R*) given by (9) with *R*_0_ = 0.4*a* and *p* = 1/2. The right hand plot shows the corresponding index, *N*(*R*) necessary to reproduce the behaviour of the Eaton lens, calculated from a numerical integration of (12).

The obvious flaw in the above theory is that the waves will be scattered from the join between the conical surface and the plane, which will spoil the function of the device. However this is not a serious obstacle, and the join between the plane and the cone can be made smooth with the consequence of a slight change in the form of the refractive index profile. For example, we could change the conical surface so that it smoothly joins the plane as in the case below, 
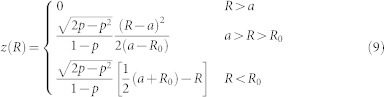
with the tip being of the required angle to mimic the singularity in the desired index profile, *n*(*r*). To determine the additional index profile *N*(*R*) required so that the device behaves as the required planar profile, *n*(*r*), we again equate length elements in the two systems, 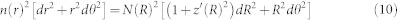
Equating the angular length elements then gives, 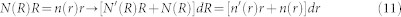
The same procedure applied to the radial length element then gives—after an application of (11)—the differential equation satisfied by *N*(*R*), 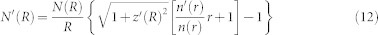
Given that we know *n*(*r*), *z*′(*R*) and the relationship between *r* and *R*, (12) can be numerically integrated to find the required *N*(*R*) for any smoothed surface, such as (9). In this manner we can remove any scattering that occurs as the waves enter onto the conical part of the surface. Figure 1 shows an example of the *N*(*R*) necessary to reproduce the behaviour of the Eaton lens, calculated from the *z*(*R*) given by (9).

## Results

**Figure 2:**
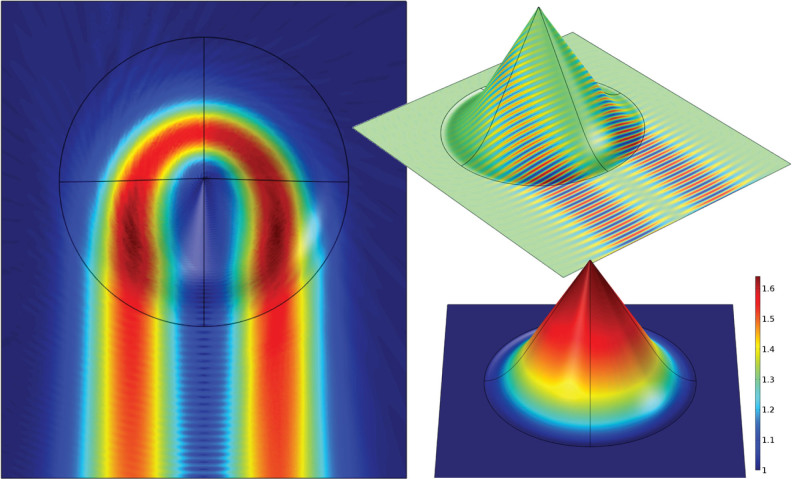
Finite element modelling of a 2D implementation of the Eaton lens (5) using a sculpted parallel plate waveguide. The device is excited using a planar narrow Gaussian beam with a wavelength of 1/10th of the radius of the deformed region offset to one side of the device. The left panel shows a top–down view of the time averaged field within the device, clearly showing the desired function of retro–reflection. The top right panel shows the instantaneous z–component (normal to the average plane of the device) of the electric field on an isometric projection of the device, where we can see the role of the tip in redirecting the incoming waves. The bottom right panel shows the required index, *N*(*R*) that must be present in the deformed region of the waveguide.

**Figure 3:**
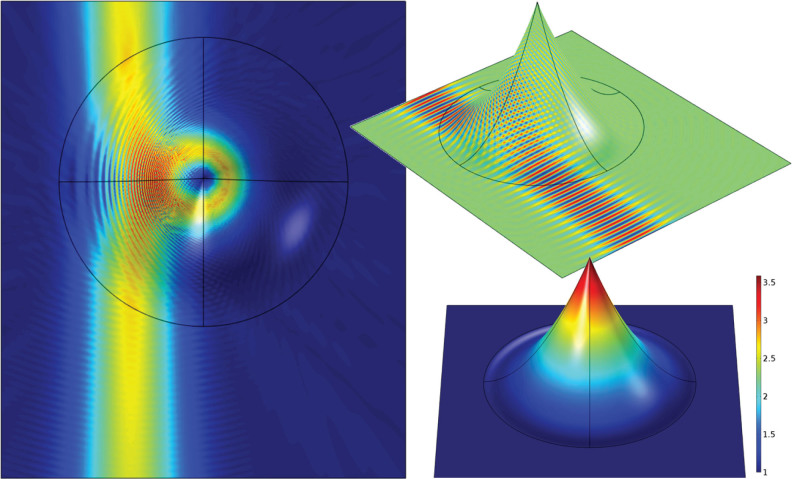
Modelling of the invisible sphere (7), with panels arranged as in figure 2. The left panel shows that the incoming beam wraps in a full circle around the tip of the cone, leaving a moderately field–free region in the centre, and then exits parallel to the incident beam.

To confirm the theory outlined above we have performed full wave simulations of the three devices in a parallel plate waveguide implementation that mimics the propagation of waves upon a surface (using Comsol Multiphysics). The waveguide is shaped into a conical profile that smoothly joins the plane, in the manner specified by (9), and ensuring that *a* – *R*_0_ is always much greater than the wavelength. It is taken to be thin enough (one tenth of the free space wave–length) so that any incoming power is only coupled into the fundamental TM mode. A dielectric is placed within the region between the plates to obtain the necessary index profile, 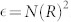. Figures 2 and 3 show simulations of an Eaton lens and an invisible sphere, and show that they perform their function exactly as expected. However, rather than the singular refractive index profiles of the originals, these conic devices require refractive index contrasts of only 1.6 and 3.6 respectively.

**Figure 4:**
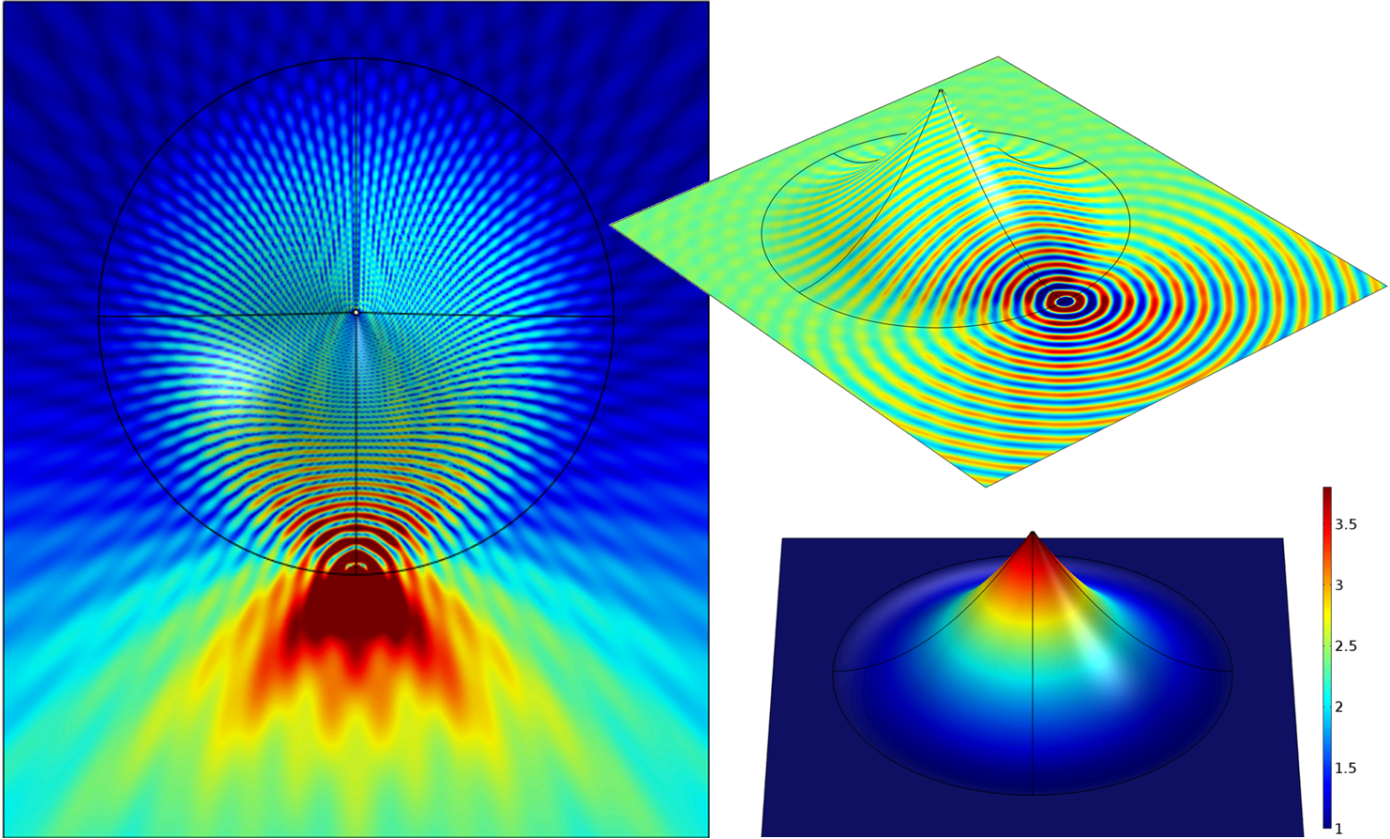
Modelling of the ‘monopole’ lens (6), with panels arranged as in figure 2. In this case the device functions as the generalized Maxwell fish eye profile (6) for the case of *M* = 2, and is excited by a point dipole located at the perimeter of the deformed region. This device is sometimes referred to as a ‘monopole’ lens, given its property of refocussing the outgoing waves back on the source, rather than at a separate point. The operation of the lens is frequency dependent due to interference within the device. Here the wavelength has been chosen to best demonstrate the ‘monopole’ nature of the device.

Figure 4 shows an implementation of the ‘monopole’ lens, which is the *M* = 2 case of the generalized Maxwell Fish Eye (6). The ‘monopole’ lens has the function of redirecting the outgoing rays from a point source placed on its perimeter back onto the source, and is achieved here with a refractive index contrast of roughly 3.7. It should be noted that the wave–nature of the system is evidenced by interference within the lens, and results in a frequency dependence in its operation. Here we have chosen a frequency which best demonstrates the properties of the device.

The theory also applies to the propagation of electromagnetic surface waves, such as surface plasmons or spoof surface plasmons. Indeed, a similar technique has already been applied as a means to conceal bumps in a surface from surface wave propagation^23^. However, in this case it is apparent that the devices need to be many wavelengths in size due to the effect of the mean curvature of the surface on the wavelength of the surface waves^24^,^25^. In the supplementary material we apply the WKB approximation to solve the wave equation for surface waves propagating on a cone, showing that the expected geometrical optics limit becomes a good approximation for large devices.

## Conclusions

We have shown that, when waves are restricted to propagate along a surface, previously impractical graded–index devices, specifically those that exhibit singularities in their refractive index profiles, become feasible. By deforming the surface one modifies the path length of a ray in a similar manner to that achieved by modifying the refractive index, and a simple ‘tip’ in the deformed surface mimics a singularity in the refractive index. We interpret this equivalence as being analogous to the transmutation of singularities in three dimensional optical instruments via a coordinate transformation^10^,^11^, although in our case the medium remains isotropic. Using this finding we have redesigned the Eaton lens, the invisible sphere and the generalized Maxwell fish eye profile—all of which exhibit singularities in their refractive index profiles—as sculpted surfaces with index contrasts of at most 3.7, and demonstrated their functionality in full wave simulations. Given that the invisible sphere is a basic element of sub–luminal invisibility cloaks^26^, this approach may be a step towards a 2D realization of these interesting, but extreme devices.

## Author Contributions

S.A.R.H. did the theory and wrote the main manuscript, I.R.H., R.C.M. and O.Q.T. did the simulations and edited the manuscript, and I.R.H. and R.C.M. prepared the figures. All authors reviewed the manuscript.

## Supplementary Material

Supplementary Information Removing singular refractive indices with sculpted surfaces (Supplementary information)
