# Supplementary material for: Removing singular refractive indices with sculpted surfaces
Source: Sci Rep. 2014 May 2;4:4876. doi: 10.1038/srep04876 (PMC4007085; doi:10.1038/srep04876)
Supplement: Supplementary Information — Removing singular refractive indices with sculpted surfaces (Supplementary information) [file srep04876-s1.pdf]

Removing singular refractive indices with sculpted surfaces (Supplementary information)

S. A. R. Horsley and I. R. Hooper
Department of Physics and Astronomy, University of Exeter, Stocker Road, Exeter, EX4 4QL, United Kingdom

R. C. Mitchell–Thomas
School of Electronic Engineering and Computer Science, Queen Mary University of London, London E1 4NS, United Kingdom

O. Quevedo–Teruel
School of Electrical Engineering, KTH Royal Institute of Technology, SE-10044, Stockholm, Sweden

WKB theory of surface waves on a cone

The main text illustrates a waveguide implementation of our theory, which is the simplest case. Here we demonstrate that the theory also applies to surface waves, provided that the device is significantly larger than the free space wavelength of the waves of interest. Here we find a general solution for surface waves moving on a cone upon which there is a radially symmetric refractive index profile. This solution is valid in the WKB approximation.

For simplicity we ignore the effects of polarization and consider a scalar wave theory for a monochromatic field, φ that obeys the wave equation,

$$\left[\boldsymbol{\nabla}^2 + k_0^2\right]\varphi = 0 \tag{1}$$

where k_0 is the free space wave–vector, and we use a spherical polar system of coordinates (r,θ,ϕ), where the surface $\theta=\theta_0$ is the surface of the cone described in the main text. A surface impedance boundary condition is used to describe the behaviour of the wave on the surface of the cone,

$$\left[\frac{1}{r}\frac{\partial\varphi}{\partial\theta} = \kappa(r)\varphi\right]_{\theta=\theta_0} \tag{2}$$

which states that the field decays in the region of space above the surface. The impedance is varied as a function of r to control the local wavelength of the surface waves in the manner required by the quantity $N(R)$ given in the main text ($R = r\sin(\theta_0)$). To solve (6) subject to (7) the field is written in the following form,

$$\varphi = ae^{iS} \tag{3}$$

where the amplitude and phase are both expanded as an asymptotic series in inverse powers of the free space wave–vector times the size of the device. Inserting the expansion (8) into (6) and (7), we obtain the following relations for the amplitude and phase, which are valid to leading order,

$$(\boldsymbol{\nabla}S)^2 = k_0^2 \tag{4}$$

$$\boldsymbol{\nabla}\cdot\left(a^2\boldsymbol{\nabla}S\right) = 0 \tag{5}$$

$$\left.\frac{\partial S}{\partial\theta}\right|_{\theta=\theta_0} = -i\kappa(r)r \tag{6}$$

$$\left.\frac{\partial a}{\partial\theta}\right|_{\theta=\theta_0} = 0 \tag{7}$$

In order to satisfy the boundary condition (11), the phase S and amplitude are written as expansions around $\theta=\theta_0$,

$$S(r,\theta) = S_0(r) - i\kappa(r)r(\theta-\theta_0) + \frac{1}{2}(\theta-\theta_0)^2 S_2(r) + l\phi \tag{8}$$

$$a(r,\theta) = a_0(r) \tag{9}$$

where l is the angular momentum of the mode around the axis of symmetry. The term a_1 is zero, due to (12). We do not look to determine a further than first order in $\theta - \theta_0$. Inserting (13) into (9) and equating like powers of $\theta - \theta_0$ gives,

$$S_0(r) = \int^r \sqrt{k_0^2 + \kappa(x)^2 - \frac{l^2}{x^2 \sin^2(\theta_0)}} dx \tag{10}$$

$$S_2(r) = \frac{i \cot(\theta_0) l^2}{\kappa(r) r \sin^2(\theta_0)} - r\sqrt{k_0^2 + \kappa(r)^2 - \frac{l^2}{r^2 \sin^2(\theta_0)}} \left(\frac{\kappa'(r) r}{\kappa(r)} + 1\right) \tag{11}$$

Expression (15) is the part of the phase that is relevant in the $k_0 \to \infty$ limit, with the effective index being given by $\sqrt{1 + \kappa(r)^2/k_0^2}$. Expression (16) represents changes in both the phase and the decay of the field away from the surface, including scattering into radiation. Having determined the phase, we insert (14–16) into (10) in order to obtain an expression for the amplitude, $a(r)$,

$$a_0(r) = \frac{1}{r\sqrt{S_0'(r)}} \exp\left(\frac{1}{2}\int^r \frac{1}{x^2 S_0'(x)} \left[\cot(\theta_0) i\kappa(x) x - S_2(x)\right] dx\right) \tag{12}$$

On the surface of the cone, $\theta = \theta_0$ the field therefore takes the form,

$$\varphi(r, \theta_0, \phi) \sim \frac{1}{r\sqrt{S_0'(r)}} e^{\frac{1}{2}\int^r \frac{1}{x^2 S_0'(x)}\left[\cot(\theta_0) i\kappa(x) x - S_2(x)\right] dx \pm i S_0(r) + il\phi} \tag{13}$$

where the particular combination of incoming and outgoing waves is determined by an analysis of the behaviour of the waves around the turning point [**?**]. There are evidently additional contributions to the phase of φ which were not taken into account in the theory we presented in the main text, where the implicit assumption was that the phase was given by S_0. Of these additional terms, the leading order contribution is given by,

$$\Delta S = \frac{\cot(\theta_0)}{2} \int^r \frac{\kappa(x)}{x\sqrt{k_0^2 + \kappa(x)^2 - \frac{l^2}{x^2 \sin^2(\theta_0)}}} dx \tag{14}$$

which reduces to $\kappa \cot(\theta_0) \log(k_0 r)/2\sqrt{k_0^2 + \kappa^2}$ when the impedance is uniform and $l = 0$, identical with the result of Lyalinov (see ref 21.). This additional contribution to the phase, (19) is proportional to the integral of the mean curvature of the cone, $M(r) = |\cot(\theta_0)|/2r$, and is small in the region where $k_0 r \gg 1$. Including this additional contribution, the magnitude of the gradient of the phase is $\sqrt{k_0^2 + \kappa(r)^2(1 + \cot(\theta_0)/\kappa(r) r)}$. Thus in the region where $\cot(\theta_0)/\kappa(r) r \ll 1$, the device ought to function according to the predictions of geometrical optics.
